# Why People Harm the Environment Although They Try to Treat It Well: An Evolutionary-Cognitive Perspective on Climate Compensation

**DOI:** 10.3389/fpsyg.2019.00348

**Published:** 2019-03-04

**Authors:** Patrik Sörqvist, Linda Langeborg

**Affiliations:** ^1^Department of Building Engineering, Energy Systems, and Sustainability Science, University of Gävle, Gävle, Sweden; ^2^Department of Occupational Health and Psychology, University of Gävle, Gävle, Sweden

**Keywords:** climate change, moral accounting, balancing heuristic, natural selection, compensatory green beliefs, negative footprint illusion, evolutionary-cognitive perspective

## Abstract

Anthropogenic climate changes stress the importance of understanding why people harm the environment despite their attempts to behave in climate friendly ways. This paper argues that one reason behind why people do this is that people apply heuristics, originally shaped to handle social exchange, on the issues of environmental impact. Reciprocity and balance in social relations have been fundamental to social cooperation, and thus to survival, and therefore the human brain has become specialized by natural selection to compute and seek this balance. When the same reasoning is applied to environment-related behaviors, people tend to think in terms of a balance between “environmentally friendly” and “harmful” behaviors, and to morally account for the average of these components rather than the sum. This balancing heuristic leads to compensatory green beliefs and negative footprint illusions—the misconceptions that “green” choices can compensate for unsustainable ones. “Eco-guilt” from imbalance in the moral environmental account may promote pro-environmental acts, but also acts that are seemingly pro-environmental but in reality more harmful than doing nothing at all. Strategies for handling problems caused by this cognitive insufficiency are discussed.

## Introduction

The environmental impact of one’s own behavior is difficult to grasp, partly because issues related to climate change are perceived as psychologically distant (cf. Spence et al., 2012). When people try to act in environmentally friendly ways, they often in fact do further harm to the environment. They might purchase some extra groceries because the groceries are “eco-labeled”; think that they can justify taking the airplane abroad for vacation because they have been taking the bicycle to work; and think that they can skip recycling their waste because they started having meat-free Mondays. Entire economic systems have been built on the same principle. Companies, private persons, and even nations, trade carbon offsets within the European Union Emission Trading Scheme, whereby they compensate emission rates with financial means. Although interventions in developing countries create some climate gains from the system, the system may also license irresponsible behavior for people prepared to pay for it. Ideas associated with “climate compensation” (e.g., planting trees, trading emission rates or supporting green projects to compensate for environmentally harmful behavior) can hence be found in the context of both local and global decision making. The purpose of the present paper is to outline a theoretical perspective on the evolutionary basis of the psychology that underpins attempts to compensate for unsustainable behavior.

## The Evolutionary Basis of Human Cognition

From an evolutionary psychology perspective, the mind can be seen as a collection of evolved adaptations; people think and behave the way they do because it has given them advantages in the process of natural selection. It is assumed that the human brain structure has been shaped by evolution, which in turn influences human cognition. One such example is hemispheric lateralization, whereby it is easier for most people to perceive speech that enters the right ear (Tervaniemi and Hugdahl, 2003).

The evolutionary perspective also assumes that evolution has shaped specific, recurring thought-patterns or mental heuristics within the human mind. A heuristic is a mental tool or guiding rule, designed to solve a specific goal (Gigerenzer, 2001). An example of such an adaptive heuristic is “availability” (Tversky and Kahneman, 1974); the tendency to think that an occurrence (e.g., a natural disaster) is more likely to happen when the memory of such an occurrence is easily accessible (as after recent reports of an earthquake on the news). Another example is “anchoring”; the tendency for estimates (e.g., of future global temperatures) to fall relatively close to available anchor points (e.g., a proposed future global temperature suggested by someone else; Joireman et al., 2010). Heuristics make information processing and decision-making fast, frugal and computationally inexpensive. They are also largely successful when applied to the type of problem they are supposed to solve. When the human brain confronts a task it is not well adapted to, however, it applies heuristics designed for other purposes. This mismatch often results in erroneous thinking (Gilovich et al., 2002), such as people being more likely to believe in global warming on hot days (Zaval et al., 2014).

## An Evolutionary-Cognitive Perspective on Sustainable Behavior

Evolutionarily speaking, problems associated with climate change and the environmental impact of one’s own behavior are novel. Moreover, the relationship between behavior and consequence in the context of climate change and environmental impact is unclear because of the large temporal and geographical distances. Because of this, people are not adapted to the challenges of climate change (Griskevicius et al., 2012), and consequently there are plenty of evolutionary adaptions and cognitive heuristics which influence the way people fail to understand human-environmental interactions accurately (Gifford, 2011; van Vugt et al., 2014; Lewandowsky, 2016; Sörqvist, 2016). Hence, unsustainable behavior often has an evolutionary basis (Griskevicius et al., 2012; van Vugt et al., 2014). For example, people tend to value personal over collective rewards. Environmental problems are often global problems that have to be dealt with through collaboration, but this collaboration is difficult as long as people must give up personal gain in favor of collective rewards. Similarly, people tend to prefer immediate over delayed rewards, which inhibits the transformation to a more sustainable lifestyle among the general public, since the temporal distance between our behavior today and future environmental gains is stretched over generations.

There are also cognitive biases specifically associated with group processes and social behavior (Engler et al., 2018). The sustainability effects of these group biases may outweigh biases on an individual level. For example, people tend to favor their own group over other groups (i.e., the in-group/out-group bias). In view of this global and international nature of climate change issues, the tendency to favor one’s own group may prevent acceptance of policies that constrain people who belong to one’s in-group in favor of people in the out-group. With this in mind, we now turn to the evolutionary basis for the (often misdirected or futile) attempts to compensate for environmentally negative behavior.

### An Evolutionary-Cognitive Perspective on Attempts to Compensate for Unsustainable Behavior

Problems associated with social interaction and various forms of social exchange have been particularly important to master for successful adaptation. Consequently, rules governing social exchange has shaped human cognition, heuristics, biases, and reasoning abilities (Cosmides, 1989; Tooby and Cosmides, 1996; Hoffman et al., 1998; Kiyonari et al., 2000; Yamagishi et al., 2007; Cosmides et al., 2010). One aspect that might be particularly important, for our purpose here, is the rules governing the balance between giving and receiving favors. People expect reciprocity in interpersonal relationships – when they give something, they generally expect something in return, and when they get something they feel obliged to return the favor. Lack of balance in give-and-receive transactions of a relationship makes people sad and compromises health and wellbeing (Buunk and Schaufeli, 1999). Neglecting the balance in a relationship by receiving more than one gives can lead to shame and guilt, and giving more than one receives can lead to anger. However, the balance can be restored when the one in debt do what is necessary to compensate for past transgressions (Xu et al., 2011).

Because of the importance of social exchange during human evolution, natural selection has shaped human cognition to compute and seek balance in social exchange efficiently. Specifically, natural selection has made this kind of moral accounting important, and formed a balancing heuristic that simplifies cognition concerning interpersonal cooperation by calculating the balance in social transactions. This balancing heuristic still influences the way people think today. For example, people often take action to maintain balance between “good” and “bad” deeds – actions that can be observed in human moral decision-making (Sachdeva et al., 2009). Morally righteous and unrighteous decisions appear to be mentally accounted for as if they balance each other out. For example, prior good deeds can “license” latter choices of a more self-indulgent character (Khan and Dhar, 2006).

Moral accounting and balance seeking behavior not only apply to social relationships, they seem to apply to environmental issues as well. Some people are more likely to cheat and steal after purchasing “eco-friendly” products (Mazar and Zhong, 2010), probably because they feel licensed to do so since they morally account the “eco-friendly” choice as a “good” deed. Moral licensing has also been observed in the context of cooperation for the good of the environment (Sachdeva et al., 2009). When people are reminded of deeds they have done that they know were harmful to the environment, they might be left with a feeling of “eco-guilt” (Mallett, 2012). People who experience eco-guilt seek pro-environmental actions to compensate for this guilt and restore balance. In their search for such a balance, people are inclined to believe in “quick-fixes,” because they want to re-establish the moral balance and escape the guilt as quickly and easily as possible. In the same way that cognitive dissonance may make people change their attitudes to reduce inconsistencies between attitudes and behavior, people may also change their evaluation of past environmentally burdening behaviors and future environmentally friendly behaviors to restore the balance in their relationship with the environment. For instance, people are more likely to sign a petition addressing environmental issues after viewing evidence of human-caused environmental damage (Rees et al., 2015). Hence, the balancing heuristic of the moral accounting seems to have been generalized to human-climate interactions although such interactions are evolutionary novel.

In social exchange, allowing give-and-take transactions to balance each other out works well to maintain well-functioning cooperation. However, the same balancing rule is not appropriate to apply to “environmentally friendly” and “harmful” behavior. When people experience a negative imbalance from having done something harmful to the environment, they may actively seek an opportunity to do something good for the environment to restore the balance. However, the environment is a complex system of processes that does not respond like a person in a reciprocal relationship. Also, and foremost, environmentally harmful behavior can neither be compensated for, restored nor undone. While the tension in a relationship, caused by a harmful action to another person, can be restored by compensation without leaving permanent changes in either person, harmful actions on the environment have permanent consequences. Flying adds to an individual’s total environmental burden, no matter how many meat free Mondays that individual has. Still, “compensatory green beliefs” are widespread in the general population (Kaklamanou et al., 2015). The balancing heuristic may make people purchase more “eco-labeled” food in order to do something good for the environment, but the best thing for the environment would of course be to consume less overall. Consuming more of something is never the best way to reduce one’s own environmental impact, even if the produce is marketed as “environmentally friendly.” In the same spirit, attempts to cancel the guilt from “harmful” deeds, by avoiding taking too hot showers, or driving vehicles that run on “sustainable fuel” might make people feel good about themselves. However, the behavior can cause even more harm to the environment, if showers instead go on for longer and people take the car to work more often (cf. rebound effects; Chitnis et al., 2013).

The balancing heuristic is not only applied when people reason about their own behavior and choices – it also generalizes to items and objects. When so-called “environmentally friendly” items are added to a set of “conventional” items, people believe the environmental impact of the whole set is reduced. For instance, people intuitively think the environmental burden of a hamburger and an “organic” apple in combination is lower than the environmental burden of the hamburger alone (Gorissen and Weijters, 2016). People mentally account for the “environmentally friendly” and the more “harmful” objects as if the objects balance each other out rather than sum up together (Holmgren et al., 2018a,b). This averaging principle leads to quantity insensitivity with regard to the amount of “environmentally friendly” objects in the set. For example, people tend to think that the environmental burden of a car pool remains the same when hybrid cars are added to the pool, as if numbers do not matter as long as the cars are seemingly friendly to the environment (Kim and Schuldt, 2018). One interpretation of this is that the balancing heuristic makes people think that the climate friendlier objects compensate for the more harmful ones, in the same manner as it does for evaluations of people’s own deeds.

### Interventions to Overcome the Sustainability Problems That Follow From the Balancing Heuristic

The complexity of the problem is too large for people to understand the effect of their consumer choices, traveling behavior, recycling efforts, and lifestyles on climatic change. Without access to more detailed information, such as life cycle analyses of the products in the grocery stores for instance, people apply the balancing heuristic to guide intuitive judgments and decision-making toward what they think is an overall averaged environmentally friendly behavior. People who experience guilt, from environmentally questionable behavior, will seek what they think are sustainable behaviors as an instrument for restoring moral balance. The balancing heuristic may lead people into believing that the more they do of something “environmentally righteous,” the more environmentally friendly they are—since they build up their moral account (cf. Sachdeva et al., 2009). However, although buying a bundle of eco-labeled bananas is better for the environment than buying the same amount of conventionally grown bananas, it is worse than not buying any bananas at all.

One way to help people make more sustainable decisions on the individual level would be to give consumers feedback on the carbon footprint of the wares they are about to purchase, for example by taking advantage of self-scanning systems, where customers scan their products themselves before paying for them. In addition to the accumulated price with each product, the system could also provide the customers with an accumulated carbon footprint estimate of their wares. That way, the costumers receives immediate feedback saying that “eco-labeled” products do not reduce but add to the accumulated carbon footprint of what they are buying. Giving direct and concrete feedback about the effect of consumer choices can work in an informational way as well as in a way that nudges people to make better choices (Linder et al., 2018). Simple aspects of the design of public places, like a paper towel dispenser that shows a green map of Africa, which slowly fades away for each towel dispended, can affect the choice architecture and thus promote environmentally friendly behavior.

Another approach would be to run information campaigns that make people aware of the misleading concepts of “climate compensation” and “environmentally friendly.” The balancing heuristic makes people susceptible to the influence of media communication and policymaking that tell people how to behave (Leiserowitz, 2006; Moser, 2009) and to marketing devices such as “moral labeling” (de Pelsmacker et al., 2005; Yiridoe et al., 2005). Words such as “environmentally friendly,” “eco-friendly” and “ecological” run the risk of establishing a public view that objects, behaviors and decisions with these labels are “good” rather than “less bad” for the environment (Holmgren et al., 2018b).

To overcome the balancing heuristic as a cognitive barrier to sustainability, we need to make people deal with the source of the problem instead of seeking ways to compensate for it, in part through education and information campaigns. The cognitive barriers are sometimes hard to break down, however, because people are not prepared to spare what is needed to deal with the source of the problem. Population growth is one of the major driving forces of future climatic change and legislations to prevent population growth would mitigate climatic changes, but such legislations are likely to be met with both ethical, political, and religious arguments (cf. van Vugt et al., 2014).

Being eager to avoid environmental imbalance and eco-guilt may not only make people ready to believe in environmental “quick-fixes.” Importantly, it may also affect people’s readiness to perceive an imbalance, or guilt, in the first place. Realizing the environmental impact of one’s own behavior is challenging, as much emotionally as cognitively. The “ostrich effect” – the tendency to selectively reject available inconvenient information, for example by ignoring the environmental impact of one’s own behavior, is a way of avoiding guilt and moral imbalance in the first place. This tendency makes it important to inform the public about the climate changes in the right way – to stress the severity of the problem enough to make people understand the problem, but not so much that it makes people reject the information.

The behavioral patterns that follow from the balancing heuristic influence decisions not only on the individual level but also on macro levels. Nations may justify weaponry exports to dictatorships by also enacting climate-change alleviation interventions in the third world. Companies license their carbon emissions by buying carbon offsets while also building customer loyalty and market their brand as “environmentally responsible.” Restaurants serving nothing but red meat may market themselves as “100% climate compensated” which attracts customers who wish to eat hamburgers without experiencing eco-guilt. A sharpened legislation of marketing of products, choices, as well as economic devices for emission regulation as if they are “environmentally friendly” alternatives, is necessary to deal with this problem. The present jurisdiction governing these devices do not fully consider their psychological consequences. Calling a hamburger restaurant “100% climate compensated,” for example, may deceive people into believing that eating dinner at that restaurant has no environmental burden. Also, things that are gentle for the closest environment (like organically produced meat) are not necessary gentle on the climate, which calls for more precise climate related labeling of products. A stricter legislation of marketing devices and an obligatory carbon footprint estimate of products could be one way to better guide people’s behavior (cf. Steiner et al., 2017), companies and nations away from environmentally harmful behavior they do when they try to do good for the environment.

## Conclusion

The proposed framework in this paper suggests that several examples of unsustainable behavior and effects (negative footprint illusions, rebound effects, compensatory green beliefs, quantity insensitivity, etc.) have their roots in mental heuristics shaped by natural selection to handle social exchange. We have tried to show how moral accounting and the balancing heuristic, apparently present in social exchange processes, can explain how people and decision makers think and act in response to environmental and climatic change issues, as well as to marketing devices, pro-environmental political policies and economic systems that involve the idea of “climate compensation.” Specifically, a reason why people sometimes harm the environment although they try to do good, is that the balancing heuristic makes them believe “environmentally friendly” behavior can compensate for unsustainable behavior. The strategies proposed can hopefully help toward reducing the negative effects of this inherited cognitive handicap.

## Author Contributions

All authors listed have made a substantial, direct and intellectual contribution to the work, and approved it for publication.

## Conflict of Interest Statement

The authors declare that the research was conducted in the absence of any commercial or financial relationships that could be construed as a potential conflict of interest.
